# Supplementary material for: Vitamin D Modified DSS-Induced Colitis in Mice via STING Signaling Pathway
Source: Biology (Basel). 2025 Jun 18;14(6):715. doi: 10.3390/biology14060715 (PMC12190092; doi:10.3390/biology14060715)
Supplement: Supplementary file 1 [file biology-14-00715-s001.zip › Supplementary Materials S1: 16s-report of gut microbiota/Groups/Network/Phylum_network.pdf]

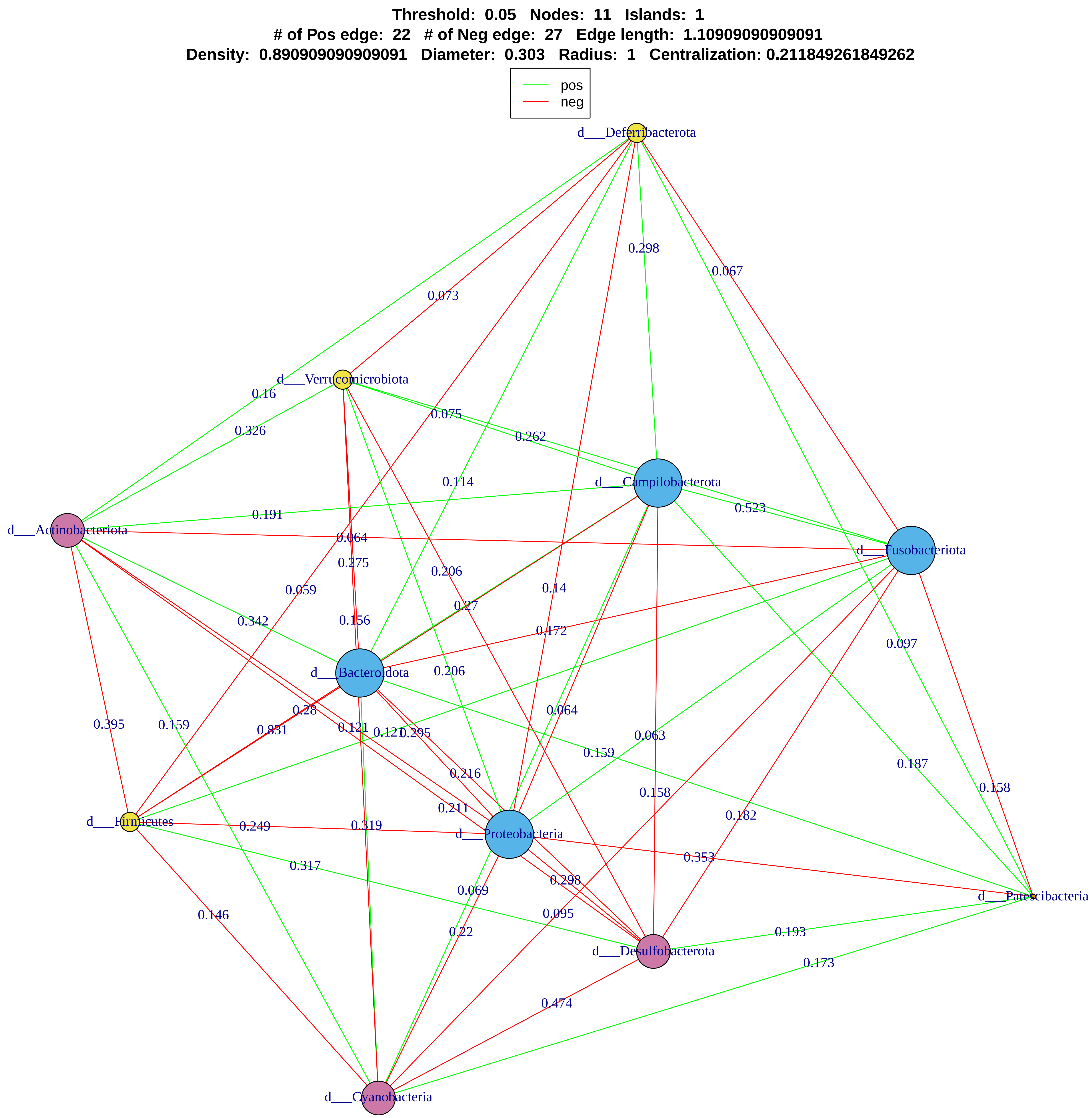

Threshold: 0.05 Nodes: 11 Islands: 1
# of Pos edge: 22 # of Neg edge: 27 Edge length: 1.10909090909091
Density: 0.890909090909091 Diameter: 0.303 Radius: 1 Centralization: 0.211849261849262
pos
neg
d___Deferribacterota
d___Verrucomicrobiota
d___Campilobacterota
d___Actinobacteriota
d___Fusobacteriota
d___Bacteroidota
d___Firmicutes
d___Proteobacteria
d___Patescibacteria
d___Desulfobacterota
d___Cyanobacteria
